# Engaging Elderly Breast Cancer Patients: The Potential of eHealth Interventions

**DOI:** 10.3389/fpsyg.2016.01825

**Published:** 2016-11-16

**Authors:** Daniela Villani, Chiara Cognetta, Davide Toniolo, Francesco Scanzi, Giuseppe Riva

**Affiliations:** ^1^Department of Psychology, Università Cattolica del Sacro CuoreMilan, Italy; ^2^Department of Medical Oncology, “G.Salvini” ASST RhodenseMilan, Italy; ^3^U.O. Oncologia Medica, Ospedale S. Giuseppe-MultimedicaMilan, Italy; ^4^Applied Technology for Neuro-Psychology Lab, Istituto Auxologico ItalianoMilan, Italy

**Keywords:** breast cancer, patient engagement, Internet interventions, eHealth, elderly patients

Breast cancer is the most common cancer in women in the world and age is the strongest risk factor for breast cancer in women. Incidence of breast cancer still increases even if at a slower rate in women aged over 50 years until age of 80 years (DeSantis et al., 2011). Actually the incidence of breast cancer represents a critical health concern in the growing aging population and requires specific evidence-based recommendations (Petrakis and Paraskakis, 2010; Biganzoli et al., 2012). From a psychological point of view, some authors found that older breast cancer patients have more difficulty in adjusting to breast cancer and related treatments than younger women and these differences resulted from several age-related factors (Park et al., 2011).

Among several treatment options, chemotherapy treatment is experienced as distressing and traumatizing and frequently considered as emblematic of the treatment and of cancer itself (Richer and Ezer, 2002), also for postmenopausal women (Browall et al., 2006). One of the worst experiences associated with the chemotherapy treatment is that of losing hair. This feeling is frequently ranked among the first three important side effects for breast cancer patients, together with nausea and fatigue (Lindop and Cannon, 2001; Carelle et al., 2002). Some studies suggest that side effects are experienced as less distressing as patients can anticipate them (Golant et al., 2003; Frith et al., 2007). This preparation constitutes a form of anticipatory coping—coping which involves the preparation for managing the stressful consequences of an upcoming event, which is likely or certain to occur (Aspinwall and Taylor, 1997).

Therefore, sustaining the engagement of breast cancer patients at different ages represents an important aim. Specifically, within the whole process of patient engagement that is composed of four incremental and evolutionary phases (Graffigna et al., 2013a,b), the period before the initation of adjuvant chemotherapy can be seen as the second phase of the breast cancer patient engagement process (*arousal phase*). On the one hand, the diagnosis is still a recent event (*blackout phase*) and the management of the emotional reactions is still difficult. On the other hand, to increase knowledge about the treatment and its related side effects and develop strategies to cope with anxiety (*adhesion phase*) represents an important challenge that contributes in maintaining future outcomes of the cancer treatment (Su et al., 2005) and in recapturing a positive life planning oriented to the future.

Age-appropriate patient education interventions might need to be designed and realized to prepare older women for the social, physical, function and treatment-related effects of breast cancer and thus reducing their anxiety and increasing their control over the situation (Treacy and Mayer, 2000; Seçkin, 2011). eHealth interventions allow to develop integrated, sustainable and patient-centered services, to promote and enhance health and to augment the efficacy and efficiency of the process of healthcare (Eysenbach, 2001; Graffigna et al., 2014; Barello et al., 2016). As investigated by Fogel et al. (2002), age, length of time since diagnosis, and breast cancer stage are unrelated to Internet use and an increasing number of patients of any age are accessing health information on the Internet (Seçkin, 2011).

Offering Web support as part of regular care can be a powerful tool to help breast cancer patients manage their illness (Ventura et al., 2013). Recently, several studies showed the efficacy of Web-based illness management systems, containing components for symptom monitoring, tailored information and self-management support, compared to usual care (Børøsund et al., 2014) in promoting emotional processing (Baker et al., 2011) and reducing depression and anxiety levels (Yun et al., 2012). A recent review suggests a positive relationship between the use of Internet - or interactive computer-based education program - and the knowledge of breast cancer patients; this relationship also has a positive effect on patient satisfaction (Ryhänen et al., 2010). However, Internet educational programs available for breast cancer patients are still rare and mostly focused on increasing patients' knowledge, focusing more on “basic details” related to the disease and information about procedures rather than on diagnosis, treatment, recovery, and quality of life (Warren et al., 2014).

Among the few eHealth interventions aimed to improve the women well-being, it is possible to describe different approaches. One includes the use of personal websites to improve the emotional wellbeing of breast cancer women by helping them to construct a narrative of their experience, express emotions, and receive the social support they need, particularly from friends and extended family (Harris et al., 2015). A second is aimed to enhance social support through online peer support interventions, and recently older women reported that they receive more benefits from using online support groups especially in regard to feeling in control of their health and feeling less distressed than younger women (Seçkin, 2011). A third proposes coping skills trainings aimed to take under control patients' affective state. With this aim, Owen et al. (2005) employed a randomized controlled design to pilot the efficacy of a self-guided coping skills training and support intervention provided over the Internet. Treatment participants showed a trend toward greater improvement in emotional well-being compared to control participants. With a similar approach, recently, Villani et al. (2016) developed a 2 weeks eHealth protocol based on Meichenbaum's Stress Inoculation Training (SIT) (Meichenbaum, 1985) intervention for helping elderly women undergoing chemotherapy to cope with impeding hair loss and other treatment side effects. The protocol was composed by three phases coherent with the general SIT objectives (Serino et al., 2014): (1) increasing knowledge about the stress process, (2) developing self-regulation skills and (3) helping individual to use the acquired coping skills in real contexts. SIT has been already applied in other studies to cancer patients and appeared beneficial in altering anxiety-related behaviors (Moore and Altmaier, 1981).

To conclude, design and develop eHealth interventions for elderly breast cancer patients represents a challenge for future interventions. These could be particularly helpful for older women who for medical, geographic, and/or social reasons, find themselves isolated and could have difficulties in accessing to other psychological services. Thus, first as elderly is not an homogenous group, different sociodemographic characteristics but also individual characteristics, such as personality traits and computer self-efficacy (Rockmann and Gewald, 2015), should be assessed as these could have implications on Internet adoption. Second, by considering this specific patient engagement phase, eHealth interventions can be used to manipulate the affective state of patients and help them in recovering control over their own experience by using different approaches and strategies (Villani and Riva, 2012; Carissoli et al., 2015).

Patient engagement constitutes a new frontier for health care models where eHealth could maximize its potentialities by targeting interventions to specific diseases and different phases of the life span (Riva et al., 2016).

## Author contributions

DV conceived the ideas presented in the article and took the lead role in drafting the article. CC, DT, FS, and GR assisted in drafting the article.

### Conflict of interest statement

The authors declare that the research was conducted in the absence of any commercial or financial relationships that could be construed as a potential conflict of interest.
